# Computational modeling to determine key regulators of hypoxia effects on the lactate production in the glycolysis pathway

**DOI:** 10.1038/s41598-020-66059-w

**Published:** 2020-06-08

**Authors:** Shabnam Hashemzadeh, Sedaghat Shahmorad, Hashem Rafii-Tabar, Yadollah Omidi

**Affiliations:** 1 Department of Medical Physics and Biomedical Engineering, School of Medicine, Shahid Beheshti University of Medical Sciences, Tehran, Iran; 2 Research Center for Pharmaceutical Nanotechnology, Biomedicine Institute, Tabriz University of Medical Sciences, Tabriz, Iran; 3 Department of Applied Mathematics, Faculty of Mathematical Sciences, University of Tabriz, Tabriz, Iran; 4 The Physics Branch of the IRI Academy of Sciences, Tehran, Iran; 5 Department of Pharmaceutics, Faculty of Pharmacy, Tabriz University of Medical Sciences, Tabriz, Iran

**Keywords:** Computational biology and bioinformatics, Computer modelling

## Abstract

In solid tumors, hypoxia can trigger aberrant expression of transcription factors and genes, resulting in abnormal biological functions such as altered energetic pathways in cancer cells. Glucose metabolism is an important part of this phenomenon, which is associated with changes in the functional expression of transporters and enzymes involved in the glycolysis pathway. The latter phenomenon can finally lead to the lactate accumulation and pH dysregulation in the tumor microenvironment and subsequently further invasion and metastasis of cancer cells. Having capitalized on the computational modeling, in this study, for the first time, we aimed to investigate the effects of hypoxia-induced factor-1 (HIF-1) mediated hypoxia on the magnitude of functional expression of all the enzymes and transporters involved in the glycolysis process. The main objective was to establish a quantitative relationship between the hypoxia intensity and the intracellular lactate levels and determine the key regulators of the glycolysis pathway. This model clearly showed an increase in the lactate concentration during the oxygen depletion. The proposed model also predicted that the phosphofructokinase-1 and phosphoglucomutase enzymes might play the most important roles in the regulation of the lactate production.

## Introduction

Glucose is the most important energy source for mammalian cells. Glucose metabolism is a binomial process in normal cells. At the initial phase, a glucose molecule undergoes the glycolysis process to produce two ATP, two NADH, and two pyruvate molecules. Next, the pyruvate is converted to acetyl-CoA and involved in the oxidative phosphorylation, in mitochondria, to generate 36 ATP molecules per one glucose molecule with CO_2_ and H_2_O, as byproducts^1,2^. In normal cells under the normoxic condition, about 10% of the energy requirements of the cell is provided by the glycolysis pathway, while in cancer cells and under hypoxic condition, the percentage of energy provided by the glycolysis can increase by up to 50%. This phenomenon is induced by the alterations in the functional expression and/or activity of enzymes and transporters involved in the glucose metabolism^3^.

The increase the glucose consumption and the glycolytic rate, during hypoxia, can lead to increased conversion of the pyruvate to lactate and hence its accumulation in the cytoplasm. Solid tumors can form a permissive milieu, so-called tumor microenvironment (TME), in which glucose is mostly metabolized via glycolysis. The resultant lactic acid metabolites are effluxed by various transporters, resulting in profound acidification of the extracellular fluid^4–6^. Hence, this phenomenon can activate the metalloproteinase enzymes, which in turn can degrade the extracellular matrix (ECM). Following these events, the cells are detached from the ECM and undergo some sort of epithelial to mesenchymal transition to avoid anoikis. These cells can enter the lymph/blood vessels and then extravasate and invade the neighboring cells/tissues – a process called metastasis^5,7,8^. The lactate acts as a fuel for the cells and also as a signaling molecule in tumor progression. Thus, the lactate plays a key role in cancer progression^9^.

The cells sense a decrease in the oxygen levels in particular with the accumulation of hypoxia-induced factor-1 (HIF-1) in the nucleus. As a transcription factor, HIF-1 can affect the expression of a large number of genes, including genes involved in glucose transportation, the glycolytic pathway, and the Krebs cycle^6^. Since the HIF-1 can synchronously influence several factors in the metabolic pathway, which are modulated as a network, little variations in the levels of this molecule may promote extensive effects in the metabolic pathway and the cell function that induces the lactate production^3,5,9,10^.

While many empirical studies have been performed on the effect of hypoxia on the lactate production and the molecular components of the network have been identified, biochemical kinetics and key regulators of the pathway have not been fully understood. Theoretical studies can clarify our understanding of the system complexities by data integration from experimental researches and studying the dynamics of interactions among different molecular components. In this line, the relationship between the oxygen availability and HIF-1 and also the effects of different regulators on HIF-1, in hypoxia, has been studied and mathematically modeled^11–13^. Further, the effect of changes in the concentrations of some glycolysis pathway enzymes and transporters on the production of lactate has been investigated^14^. In the present study, to determine the key regulators of hypoxia, we aimed to establish a mathematical model to demonstrate the quantitative relationship between the oxygen availability to cells and the production of lactate.

## Modeling hypoxia effect on the glycolysis pathway

**Figure 1:**
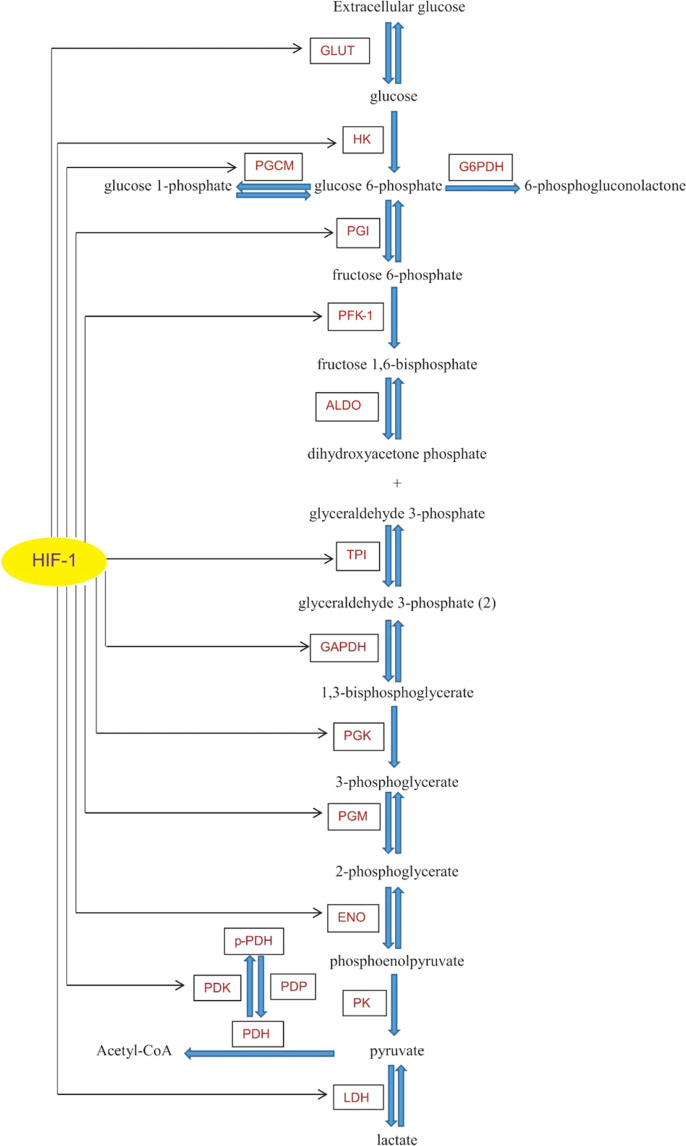
The diagram representing the molecular interactions involved in the glycolysis pathway with the sites affected by oxygen levels. GLUT: glucose transporter, HK: hexokinase, PGI: phosphoglucoisomerase, PFK-1: phosphofructokinase-1, ALDO: aldolase, TPI: triosephosphate isomerase, GAPDH: glyceraldehyde 3-phosphate dehydrogenase, PGK: phosphoglycerate kinase, PGM: phosphoglycerate mutase, ENO: enolase, PK: pyruvate kinase, PDH: pyruvate dehydrogenase, PDK: pyruvate dehydrogenase kinase, PDP: pyruvate dehydrogenase phosphatase, p-PDH: phosphorylated pyruvate dehydrogenase, LDH: lactate dehydrogenase, PGCM: phosphoglucomutase, G6PDH: glucose 6-phosphate dehydrogenase.

As shown in Fig. 1, at the beginning of the pathway, extracellular glucose (Eg) molecules enter the cell through the glucose transporters. These molecules are phosphorylated by hexokinase (HK) enzyme and converted to glucose 6-phosphate, which may face different paths, including (i) entering to the pathway of the pentose phosphate, (ii) being involved in the glycogen synthesis, and/or (iii) entering into the glycolysis pathway to meet the cellular energy demand. In the glycolysis pathway, phosphoglucoisomerase (PGI) enzyme converts the glucose 6-phosphate to the fructose 6-phosphate and the kinase activity of the phosphofructokinase-1 on the fructose 6-phosphate leads to the fructose 1,6-bisphosphate production. In the following, the sequential activity of enzymes ends with the pyruvate production. In the presence of oxygen, pyruvate is converted to the acetyl-CoA and involved in oxidative phosphorylation. Under the hypoxic condition, the lactate dehydrogenase (LDH) enzyme converts a large amount of pyruvate to lactate which is an important factor for solid tumor diagnosis. HIF-1, as an important mediator of the hypoxia effect on glucose metabolism, affects the functional expression of a large number of the enzymes involved in the glycolysis pathway (Fig. 1). Changes in the concentration of these enzymes cause changes in the levels of the metabolites and eventually in the concentration of lactate^1,3,15–23^.

Since biological systems are modulated as a network^24,25^, we assumed that all the events during hypoxia (i.e., from the glucose transportation to the initiation of metastasis) form a network of biological interactions with coordinated functions that are regulated by the HIF-1. Thus, for the first time, we have quantitatively modeled the effects of the HIF-1 on (i) the concentration and activity of all the important enzymes and transporters involved in the glycolytic pathway, and (ii) the production of lactate from the perspective of the molecular systems biology. The results of this study might illuminate our understanding of the mechanism(s) and key regulators of the glycolysis pathway. Such data may provide key information in terms of the hypoxia-mediated signaling in conditions such as initiation and progression of solid tumors that are closely associated with aberrant energy metabolism through the glycolysis pathway.

## Results

In our equations, hypoxia was introduced based on different levels of HIF-1. For a better understanding, the results were also reported based on the metabolites changes versus oxygen percentage. For this conversion, we capitalized on the relationship between HIF-1 and oxygen, which has already been reported by Qutub and coworkers^11^.

### Overview of the concentration changing in the metabolites

**Figure 2:**
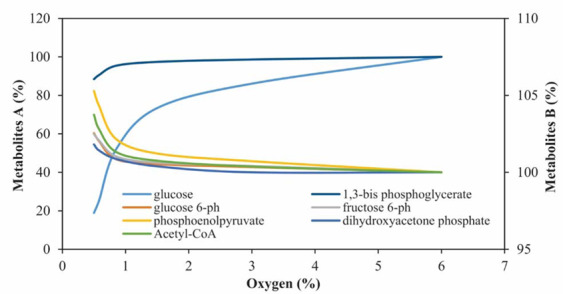
The percentage changes in the concentrations of metabolites under the influence of different levels of oxygen after 30 min. Metabolites of group A: glucose and 1,3-bisphosphoglycerate. Metabolites of group B: glucose 6-phosphate, fructose 6-phosphate, phosphoenolpyruvate, dihydroxyacetone phosphate, Acetyl-CoA.

In this work, changes in the intracellular concentrations of all metabolites of the glycolysis pathway, affected by the oxygen levels, were studied. As shown in Fig. 2, the metabolites of group A (especially glucose) were significantly reduced under the hypoxic conditions. These data are in agreement with some previous studies, which showed a decrease in the concentration of glucose during hypoxia^26,27^. The changes in the concentration of metabolites of group B were less than 5% and the remaining five metabolites did not show any changes during the reduction of oxygen levels.

### The dynamics of lactate

**Figure 3:**
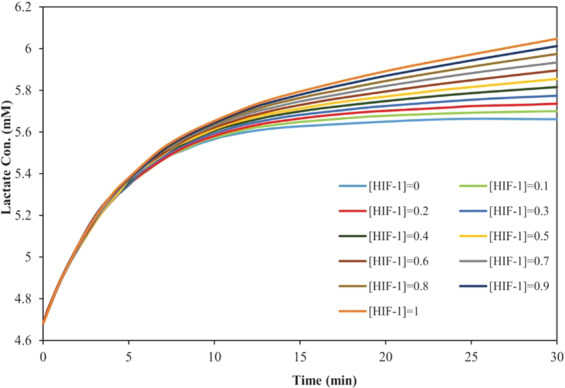
The influences of hypoxia on the lactate concentration for 30 min in different concentrations (μM) of HIF-1.

Figure 3 reveals an increase in the lactate concentration, as the final and very important metabolite of the glycolysis pathway, in response to an increase in the HIF-1 levels within 30 min. The results obtained from samples of volunteers’ blood during endurance exercises have also shown such a trend between the changes in the lactate concentration and the time at different levels of oxygen^7,28^.

**Figure 4:**
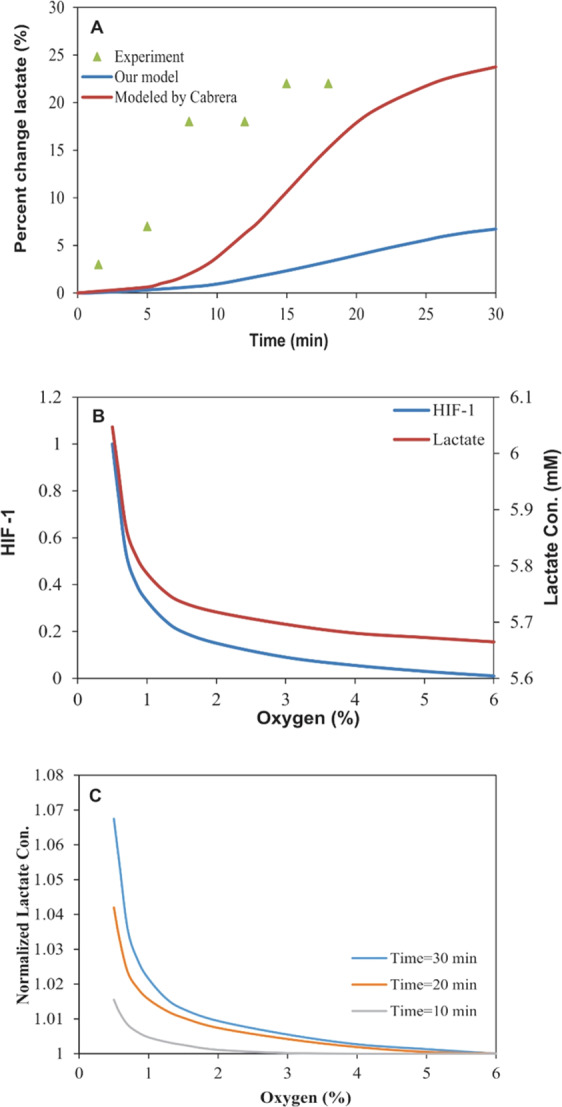
Changes in the intracellular lactate levels during severe hypoxia. (**A**) The intracellular lactate level changes during severe hypoxia in the proposed model, experimental data and Cabrera model. (**B**) The changes in HIF-1 (studied by Qutub) and the lactate concentration vs. oxygen levels after 30 min of hypoxia exposure in the proposed model. (**C**) The normalized lactate concentrations after different periods of hypoxia exposure.

Cabrera and coworkers have modeled the lactate accumulation during muscle ischemia^29^. In another study, the lactate concentration has been studied in the human brain during hypoxia^30^. Figure 4A compares the results from our model with this theoretical and experimental researches^29,30^. The trend of increase of the lactate concentration during severe hypoxia in these studies was found to be similar. As our model predicted, the influences of hypoxic conditions on the lactate production appear after about 5 min exposure to hypoxia. This delay in the appearance of hypoxia effects has already been confirmed by other computational and experimental studies^27,29,30^.

### The lactate concentration vs. oxygen levels

Figure 4B shows that the changes in oxygen levels from 6% to 0% resulted in an increase in the lactate concentration from 5.66 mM to 6.05 mM. As presented in Fig. 4B, the pattern of the correlation between the oxygen level and the lactate concentration appeared to correspond to the O_2_-HIF-1 relationship, as reported by Qutub and coworkers^11^. These findings might imply the existence of a linear relationship between the HIF-1 and the lactate levels.

Based on the results from experimental researches, there are no significant changes in the concentration of lactate with a decrease in oxygen to 6%. The concentration of lactate increases with a gentle gradient against oxygen depletion from 6% to 1.5%, then followed by an increase with a sharp slope in very low oxygen levels^31^. These experimental findings are in good consensus with the model’s predictions. The investigation of changes in the lactate levels (normalized to its value in normoxia) at different time intervals vs. oxygen changes revealed that the effects of hypoxia on the lactate levels could be increased with the increasing period of hypoxia exposure (Fig. 4C). The trend of changes in the lactate levels during hypoxia over time and also against different levels of oxygen was confirmed by the experimental studies, while the increase in the lactate levels in some experimental researches has been reported to be more than what was predicted by our model^28,31,32^.

### Sensitivity analysis

**Figure 5:**
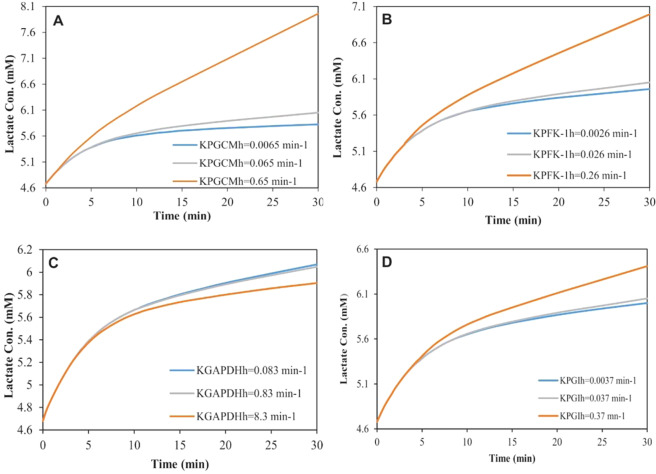
The model predictions for the lactate concentration sensitivity to (**A**) *K*_*PGCMh*_, (**B**) *K*_*PFK-1h*_, (**C**) *K*_*GAPDHh*_ and (**D**) *K*_*PGIh*_.

Sensitivity analysis was performed to investigate the effects of the *K*_*Eh*_ changes, for each enzyme, on the model’s predictions. The values of the *K*_*Eh*_ parameter for each enzyme were obtained from our calculations and the robustness of the model predictions against changes of *K*_*Eh*_ for the desired enzyme was evaluated in a range of 0.1 to 10 times of its value (Supplementary Table S1). Further, the remaining parameters were held constant. The comparison of the resulted lactate concentrations, based on *K*_*Eh*_, 0.1*K_*Eh*_ and 10*K_*Eh*_ for each enzyme, represented that the variations in *K*_*GLUTh*_, *K*_*HKh*_, *K*_*PGKh*_, *K*_*PGMh*,_ and *K*_*ENOh*_ did not show significant effects on the lactate production. Changes in the *K*_*TPIh*_, *K*_*PDKh*_, and *K*_*LDHh*_ showed a slight effect on the lactate levels. The proposed model showed that the *K*_*PGCMh*_, *K*_*PFK-1h*_, *K*_*GAPDHh*_ and *K*_*PGIh*_ most affected on the lactate production (Fig. 5).

As shown in Fig. 5, by changing the values of the *K*_*Eh*_ for each of these four enzymes, the amount of lactate produced during glycolysis was significantly changed regarding the reference values (Table S1, Supplementary Data), indicating the model sensitivity to these parameters. While increasing the *K*_*PGCMh*_, *K*_*PFK-1h*,_ and *K*_*PGIh*_ significantly accelerated the lactate production, the increase of the *K*_*GAPDHh*_ led to a decrease in the final product of glycolysis.

## Discussion

In the current study, we examined the hypoxia effect(s) on all the metabolites of the glycolysis pathway. Our results showed that the levels of glucose and 1,3-bisphosphoglycerate are in reverse correlation with the severity of hypoxia and decrease with its increase. The glucose depletion during hypoxia has already been confirmed by several experimental studies^26,27^. It seems that the main reason for this phenomenon is possibly the irreversible functions of the enzymes that can convert these metabolites. When the concentration of these enzymes increases under the influence of oxygen depletion, due to the irreversibility of the unidirectional conversion processes, respective substrate levels are reduced. Unlike lactate, other metabolites showed no significant changes. Based on our analyses, the effect of hypoxia on the enzymatic functions might be balanced for the producer and consumer enzymes of each of these substrates.

Having compared our findings with the results reported by some theoretical^29^ and experimental^30^ researches, the trend of changes in intracellular lactate concentrations seems to be similar to the changes in the lactate accumulation in blood and different tissues during hypoxia. The studies showed a delay in the appearance of the effect of hypoxia that might be attributed to the time required for the HIF-1 influence on the functional expression of enzymes and ultimately on the concentration of lactate. There was a significant increase a few minutes after the onset of hypoxia that was continued by the gentle slope within the next minutes, which indicates the trend towards a balance between the rate of production and removal of lactate. Despite this similarity, the increase in lactate predicted by our proposed model is lower than those reported by some experimental researches^28,31,32^. We suggest three assumptions to justify this difference. First, the HIF’s influence on the levels of enzymes (*K*_*Eh*_) was calculated based on the results of empirical studies carried out for more than 8 hours of hypoxia exposure, which might be different from the effects in the early stages of hypoxia (30 min in our study). Second, the hypoxia might also affect the lactate accumulation through the HIF-independent mechanisms. Third, the rate of the lactate transfer out of the cell is higher than the rate of the clearance from the blood.

Our results, from sensitivity analysis, predicted that the model might be robust over a wide range of changes in levels of more enzymes and only sensitive to changes in levels of the four enzymes, phosphofructokinase-1, glyceraldehyde 3-phosphate dehydrogenase, phosphoglucomutase, and phosphoglucoisomerase. Previously, the PFK-1 has been shown as the key regulator of the glycolysis pathway^33^, which could be a further confirmation for the high capacity of the proposed model in the prediction of molecular events during glycolysis. On the other hand, the decreased glycogen levels in different organs (e.g., brain, liver, and muscle) during hypoxia^34^ might indicate that the PGCM could provide a secondary source for the supply of energy, metabolites and the lactate production. Hence, it might play an important role in the control of lactate production. We speculate that such a phenomenon must be through the conversion of glucose 1-phosphate to glucose 6-phosphate on the glycogenolysis pathway. This could justify the low sensitivity of the proposed model to the changes in V_m_ of GLUT as glucose transporter as well as HK as the initiator enzyme of the glycolysis process.

The correlation between the results from our theoretical study and experimental researches might imply the capacity of the proposed model to explain the glycolysis process and identify the key regulators.

Upon the validation of our findings by the relevant experimental works, the regulation of the expression of the corresponding genes of these enzymes, PGCM and PFK-1, could be considered as important targets for controlling the glycolysis pathway.

## Conclusion

In the present study, we modeled the hypoxia effects on the glycolysis pathway and investigated the quantitative relationship between different levels of oxygen availability of the cell and intracellular concentration of the metabolites of the glycolysis pathway. The proposed model was found to accurately predict the decreased concentration of intracellular glucose and the increased concentration of intracellular lactate during hypoxia. The model also introduced the PFK-1 and the PGCM enzymes as key regulators for the control of the lactate production. Therefore, the local inhibition of gene expression or the function of these two enzymes, in addition to cutting off the energy supply pathway for cancer cells in solid tumors, could prevent the production of lactate and the acidification of the extracellular microenvironment in hypoxia-related diseases such as cancers.

## Methods

To investigate the relationship between the oxygen availability in the cell and the lactate production by the cell, a mathematical model was constructed based on the HIF-1 interactions with the glycolysis pathway. To model this pathway, all the involved transporters, enzymes, and metabolites were considered during the modeling. All of these factors and their interactions^1,3,15–23^ are illustrated in Fig. 1.

### Basic equations

Changes in the concentration of enzymes and metabolites, during hypoxia, were obtained using Eq. 1.

1 $$d[C]/dt=\Sigma Vp-\Sigma Vd$$

where, *d*[*C*] is the concentration changes of each the enzymes and metabolites over time (*dt*) and ∑*V*_*p*_ and ∑*V*_*d*_ represent the sum of its production rates and its degradation rates, respectively.

The influence of the HIF-1 on the concentration of each enzyme was indicated by *K*_*Eh*_, where K is the constant of the HIF-1 (h) effect on the level of a specific enzyme (E). *K*_*Eh*_ was calculated from the experimental works, based on the Mass-action law (Eq. 2).

2 $$d[CE]/dt=KEh\ast [HIF-1]$$

where, *d*[*C*_*E*_] indicates the change in the enzyme level under the influence of the HIF-1, *dt* is the period of hypoxia exposure and the [HIF-1] represents the HIF-1 level that is the criteria of hypoxia intensity.

The Michaelis-Menten equation was used as the basis, describing the kinetics of the interactions between the enzymes and the metabolites.

### Solving the equation system

An equation system was constructed based on the above basic equations. It consists of 26 ordinary differential equations (ODE), incorporating all the interactions involved in the pathway (Supplementary Information). The equation system used in this study was solved using the Mathworks Matlab software (R2015a). The ode45s solver was used to solve the set of twenty-six differential equations. This modeling aimed to quantify the effects of the oxygen level on the enzymes and metabolites involved in the glycolysis pathway and determine key regulators.

Supplementary Table S1 contains the set of initial conditions and parameters used in the current study. Most parameters were taken from the literature and two valid biological databases, including BRENDA and Sabio-RK. Others were calculated based on the referred researches or estimated by our results.

### Ethical issues

This study was ethically approved (IR.SBMU.MSP.REC.1396.753) by Shahid Beheshti University of Medical Sciences and carried out at the Research Center for Pharmaceutical Nanotechnology at Tabriz University of Medical Sciences.

## Supplementary information

SUPPLEMENTARY INFO.
