# Supplementary material for: Computational modeling to determine key regulators of hypoxia effects on the lactate production in the glycolysis pathway
Source: Sci Rep. 2020 Jun 8;10:9163. doi: 10.1038/s41598-020-66059-w (PMC7280308; doi:10.1038/s41598-020-66059-w)
Supplement: Supplementary file 1 — SUPPLEMENTARY INFO. [file 41598_2020_66059_MOESM1_ESM.pdf]

Computational modeling to determine key regulators of hypoxia effects on lactate production in the glycolysis pathway

Shabnam Hashemzadeh[1,2], Sedaghat Shahmorad[3], Hashem Rafii-Tabar[1,4*], Yadollah Omidi[2,5*]

[1] Department of Medical Physics and Biomedical Engineering, School of Medicine, Shahid Beheshti University of Medical Sciences, Tehran, Iran

[2] Research Center for Pharmaceutical Nanotechnology, Biomedicine Institute, Tabriz University of Medical Sciences, Tabriz, Iran

[3] Department of Applied Mathematics, Faculty of Mathematical Sciences, University of Tabriz, Tabriz, Iran

[4] The Physics Branch of the IRI Academy of Sciences, Tehran, Iran

[5] Department of Pharmaceutics, Faculty of Pharmacy, Tabriz University of Medical Sciences, Tabriz, Iran

*Corresponding authors:

Email: rafii-tabar@nano.ipm.ac.ir, Tehran, Iran. Fax: +982123872566;

Email: yomidi@tbzmed.ac.ir, Tabriz, Iran. Fax: +984133367929

MATLAB program

```
%This program investigates the influence of hypoxia on the production
%of glycolysis metabolites, especially lactate (y(11)). The program
%output is the enzymes and or metabolites concentration vs time, you can
%modify the program to have different levels of hypoxia (h=0:0.1:1). Hence,
%metabolites concentration can be reported vs Hif-1 or oxygen levels during
%different time intervals of hypoxia exposure. The detailed description of
%the model has been explained in the paper.



clc
close all
clear

Vmglut3=8000;                %micM/min
kmglut3=5000;                %micM
kmglut1=3000;                %micM
kcatglut1=69960;             %min-1
E0glut=0.014;                %micM
kgluth=0.0001;               %min-1
kmglut2=25000;               %micM
kcatglut2=445500;            %min-1
kdglut=0.00016;              %min-1
kmhk=40.7;                   %micM
khkh=0.003;                  %min-1
kdhk=0.002;                  %min-1
kcathk=12000;                %min-1
E0hk=0.47;                   %micM
kpgih=0.037;                 %min-1
kmpgi1=480;                  %micM
kmpgi2=272;                  %micM
kcatpgi1=125400;             %min-1
kcatpgi2=119586.8;           %min-1
kdpgi=0.00048;               %min-1
E0pgi=33.2;                  %micM
kmpfk=2000;                  %micM
kcatpfk=61200;               %min-1
E0pfk=42.5;                  %micM
kdpfk=0.00085;               %min-1
kpfkh=0.026;                 %min-1
vmaldo1=1028106;             %micM/min
kmaldo1=3;                   %micM
vmaldo2=832276.3;            %micM/min
kmaldo2=1000;                %micM
kmaldo22=2000;               %micM, for metabolite 2 in reverse direction
kmgapdh1=82;                 %micM
kmgapdh2=130;                %micM
kgapdhh=0.83;                %min-1
kdgapdh=0.00016;             %min-1
E0gapdh=1195;                %micM
kcatgapdh1=17400;            %min-1
kcatgapdh2=19.81;            %min-1
kcatpgk=29210;               %min-1
```

```
kmpgk=16.7;             %micM
E0pgk=64.4;             %micM
kdpgk=0.0013;           %min-1
kpgkh=0.3;              %min-1
kmpgm1=200;             %micM
kmpgm2=190;             %micM
kcatpgm1=60000;         %min-1
kcatpgm2=27272.7;       %min-1
E0pgm=173;              %micM
kdpgm=0.0197;           %min-1
kpgmh=0.36;             %min-1
kmeno1=16.2;            %micM
kmeno2=238;             %micM
kenoh=0.23;             %min-1
kcateno1=11160;         %min-1
kcateno2=2610.96;       %min-1
E0eno=135;              %micM
kdeno=0.00048;          %min-1
vmpk=2819725.8;         %micM/min
kmpk=1500;              %micM
kmpdk=20;               %micM
kpdkh=0.000017;         %min-1
kdpdk=0.00048;          %min-1
kcatpdk=33;             %min-1
E0pdk=0.01;             %micM
kmpdh=10;               %micM
E0pdh=27.2;             %micM
kmldh1=200;             %micM
kmldh2=11000;           %micM
kcattpi1=350000;        %micM
kcattpi2=7700092.4;     %micM
kmtpi1=870;             %micM
kmtpi2=460;             %micM
E0tpi=96.6;             %micM
ktpih=0.3;              %min-1
kdtpi=0.069;            %min-1
kldhh=0.003;            %min-1
kcatldh1=122000;        %min-1
kcatldh2=4900;          %min-1
E0ldh=1.2;              %micM
kdldh=0.0013;           %min-1
kcatpdh=105800;         %micM
kmg6pdh=77;             %micM
kmpgcm2=45;             %micM
kmpgcm1=670;            %micM
E0pgcm=28.2;            %micM
kpgcmh=0.065;           %min-1
kdpgcm=0.00016;         %min-1
kcatpgcm1=73700;        %min-1
kcatpgcm2=127220.9;     %min-1
glucose1ph=25;          %micM
Eg=10000;               %micM
vmg6pdh=120120;         %micM/min
E0g6pdh=4.4;            %micM
kAcCoA=10000;           %min-1
E0pdp=0.02;             %micM
kcatpdp=300;            %min-1
```

```
kmpdp=7.6;                %micM
h=0;                      %micM,0:0.1:1

%d[C]/dt=vp-vd
f=@(t,y)[kcatglut1*y(14)*Eg/(kmglut1+Eg)-kcatglut2*y(14)*y(1)/(kmglut2+y(1))-
kcathk*y(15)*y(1)/(kmhk+y(1))+Vmglut3*Eg/(kmglut3+Eg);...
%dy(1)/dt
kcathk*y(15)*y(1)/(kmhk+y(1))-
kcatpgi1*y(16)*y(2)/(kmpgi1+y(2))+kcatpgi2*y(16)*y(3)/(kmpgi2+y(3))-
vmg6pdh*y(2)/(kmg6pdh+y(2))-
kcatpgcm1*y(26)*y(2)/(kmpgcm1+y(2))+kcatpgcm2*y(26)*glucose1ph/(kmpgcm2+gluco
se1ph);...                      %dy(2)/dt
kcatpgi1*y(16)*y(2)/(kmpgi1+y(2))-kcatpgi2*y(16)*y(3)/(kmpgi2+y(3))-
kcatpfk*y(17)*y(3)/(kmpfk+y(3));...
%dy(3)/dt
kcatpfk*y(17)*y(3)/(kmpfk+y(3))-
vmaldo1*y(4)/(kmaldo1+y(4))+vmaldo2*y(5)/(kmaldo2+y(5))+
vmaldo2*y(12)/(kmaldo22+y(12));...
%dy(4)/dt
vmaldo1*y(4)/(kmaldo1+y(4))-
vmaldo2*y(5)/(kmaldo2+y(5))+kcattpi1*y(18)*y(12)/(kmtpi1+y(12))-
kcattpi2*y(18)*y(5)/(kmtpi2+y(5))+kcatgapdh2*y(19)*y(13)/(kmgapdh2+y(13))-
kcatgapdh1*y(19)*y(5)/(kmgapdh1+y(5));...                          %dy(5)/dt
kcatpgk*y(20)*y(13)/(kmpgk+y(13))-
kcatpgm1*y(21)*y(6)/(kmpgm1+y(6))+kcatpgm2*y(21)*y(7)/(kmpgm2+y(7));...
%dy(6)/dt
kcatpgm1*y(21)*y(6)/(kmpgm1+y(6))-kcatpgm2*y(21)*y(7)/(kmpgm2+y(7))-
kcateno1*y(22)*y(7)/(kmeno1+y(7))+kcateno2*y(22)*y(8)/(kmeno2+y(8));...
%dy(7)/dt
kcateno1*y(22)*y(7)/(kmeno1+y(7))-vmpk*y(8)/(kmpk+y(8))-
kcateno2*y(22)*y(8)/(kmeno2+y(8));...
%dy(8)/dt
kcatpdh*y(24)*y(10)/(kmpdh+y(10))-kAcCoA*y(9);...
%dy(9)/dt
vmpk*y(8)/(kmpk+y(8))-kcatpdh*y(24)*y(10)/(kmpdh+y(10))-
kcatldh1*y(23)*y(10)/(kmldh1+y(10))+kcatldh2*y(23)*y(11)/(kmldh2+y(11));...
%dy(10)/dt
kcatldh1*y(23)*y(10)/(kmldh1+y(10))-kcatldh2*y(23)*y(11)/(kmldh2+y(11));...
%dy(11)/dt
vmaldo1*y(4)/(kmaldo1+y(4))-vmaldo2*y(12)/(kmaldo22+y(12))-
kcattpi1*y(18)*y(12)/(kmtpi1+y(12))+kcattpi2*y(18)*y(5)/(kmtpi2+y(5));...
%dy(12)/dt
kcatgapdh1*y(19)*y(5)/(kmgapdh1+y(5))-
kcatgapdh2*y(19)*y(13)/(kmgapdh2+y(13))-kcatpgk*y(20)*y(13)/(kmpgk+y(13));...
%dy(13)/dt
%d[E]/dt=kEh[h]-kd[E-E0]
(kgluth*h)-(kdglut*(y(14)-E0glut));...
%dy(14)/dt
(khkh*h)-(kdhk*(y(15)-E0hk));...
%dy(15)/dt
(kpgih*h)-(kdpgi*(y(16)-E0pgi));...
%dy(16)/dt
(kpfkh*h)-(kdpfk*(y(17)-E0pfk));...
%dy(17)/dt
(ktpih*h)-(kdtpi*(y(18)-E0tpi));...
%dy(18)/dt
```

```
(kgapdhh*h)-(kdgapdh*(y(19)-E0gapdh));...
%dy(19)/dt
(kpgkh*h)-(kdpgk*(y(20)-E0pgk));...
%dy(20)/dt
(kpgmh*h)-(kdpgm*(y(21)-E0pgm));...
%dy(21)/dt
(kenoh*h)-(kdeno*(y(22)-E0eno));...
%dy(22)/dt
(kldhh*h)-(kdldh*(y(23)-E0ldh));...
%dy(23)/dt
-kcatpdk*y(25)*(y(24))^2.3/(kmpdk+(y(24))^2.3)+kcatpdp*E0pdp*(E0pdh-
y(24))/(kmpdp+(E0pdh-y(24)));...
%dy(24)/dt
(kpdkh*h)-(kdpdk*(y(25)-E0pdk));...
%dy(25)/dt
(kpgcmh*h)-(kdpgcm*(y(26)-E0pgcm))];
%dy(26)/dt
 [t, y]=ode45(f, [0, 30], [2377 1033 362 46 21 44 5 8 1.3 56 4680 43 0.18
0.014 0.47 33.2 42.5 96.6 1195 64.4 173 135 1.2 27.2 0.01 28.2]);

 %glucose
figure(1)
plot(t,y(:,1));
%glucose 6-phosphate
figure(2)
plot(t,y(:,2));
%fructose 6-phosphate
figure(3)
plot(t,y(:,3));
%fructose 1,6-bisphosphate
figure(4)
plot(t,y(:,4));
%glyceraldehyde 3-phosphate
figure(5)
plot(t,y(:,5));
%3-phosphoglycerate
figure(6)
plot(t,y(:,6));
%2-phosphoglycerate
figure(7)
plot(t,y(:,7));
%phosphoenolpyruvate
figure(8)
plot(t,y(:,8));
%Ac-CoA
figure(9)
plot(t,y(:,9));
%pyruvate
figure(10)
plot(t,y(:,10));
%lactate
figure(11)
plot(t,y(:,11));
%dihydroxyacetone phosphate
figure(12)
plot(t,y(:,12));
%1,3-bis phosphoglycerate
```

```
figure(13)
plot(t,y(:,13));
%glut
figure(14)
plot(t,y(:,14));
%hk
figure(15)
plot(t,y(:,15));
%pgi
figure(16)
plot(t,y(:,16));
%pfk
figure(17)
plot(t,y(:,17));
%tpi
figure(18)
plot(t,y(:,18));
%gapdh
figure(19)
plot(t,y(:,19));
%pgk
figure(20)
plot(t,y(:,20));
%pgm
figure(21)
plot(t,y(:,21));
%eno
figure(22)
plot(t,y(:,22));
%ldh
figure(23)
plot(t,y(:,23));
%pdh
figure(24)
plot(t,y(:,24));
%pdk
figure(25)
plot(t,y(:,25));
%pgcm
figure(26)
plot(t,y(:,26));
```

Table S1. Constants and initial conditions

Constants, initial conditions	Value (μM)	Ref.	Constants, initial conditions	Value (μM)	Ref.
[*glucose1-phosphate*]0	25	[1]	Km_{GLUT}	3000	[2]
[E_g]	10000	[3]	Km_{GLUT2}	25000	[4]
[y1]0=[*glucose*]0	2377	[5]	Km_{HK}	40.7	[6]
[y2]0=[*glucose 6-ph*]0	1033	[5]	Km_{PGI1}	480	[7]
[y3]0=[*fructose 6-ph*]0	362	[5]	Km_{PGI2}	272	[7]
[y4]0=[*fructose 1,6-bisphosphate*]0	46	[5]	$Km_{PFK\text{-}1}$	2000	[8]
[y5]0=[*glyceraldehyde 3-phoaphate*]0	21	[5]	Km_{ALDO1}	3	[9]
[y6]0=[*3-phosphoglycerate*]0	44	[5]	Km_{ALDO2}	1000	[9]
[y7]0=[*2-phosphoglycerate*]0	5	[5]	Km_{ALDO22}	2000	[9]
[y8]0=[*phosphoenolpyruvate*]0	8	[5]	Km_{GAPDH1}	82	[10]
[y9]0=[*Ac-CoA*]0	1.3	[5]	Km_{GAPDH2}	130	[10]
[y10]0=[*pyruvate*]0	56	[5]	Km_{PGK}	16.7	[11]
[y11]0=[*lactate*]0	4680	[5]	Km_{PGM1}	200	[12]
[y12]0=[*dihydroxyacetone phosphate*]0	43	[5]	Km_{PGM2}	190	[12]
[y13]0=[*1,3-bis phosphoglycerate*]0	0. 18	[13]	Km_{ENO1}	16.2	[14]
[y14]0=[*GLU*	0.014	[2,15]	Km_{ENO2}	238	[14]
[y15]0=[*HK*]0	0.47	[5]	Km_{PK}	1500	[16]
[y16]0=[*PGI*]0	33.2	[5]	Km_{PDK}	20	[17]
[y17]0=[*PFK-1*]0	42.5	[5]	Km_{PDH}	10	[18]
[y18]0=[*TPI*]0	96.6	[5]	Km_{LDH1}	200	[19]
[y19]0=[*GAPDH*]0	1195	[5]	Km_{LDH2}	11000	[19]
[y20]0=[*PGK*]0	64.4	[5]	Km_{TPI1}	870	[20]
[y21]0= [*PGM*]0	173	[5]	Km_{TPI2}	460	[20]
[y22]0=[*ENO*]0	135	[5]	Km_{G6PDH}	77	[21]
[y23]0= [*LDH*]0	1.2	[5]	Km_{PGCM1}	670	[22]
[y24]0=[*PDH*]0	27.2	[5]	Km_{PGCM2}	45	[22]
[y25]0=[*PDK*]0	0.01	[23]			
[y26]0=[*PGCM*]0	28.2	[5]			
H	0:0.1:1	[24]			

Table S1. Constants and initial conditions (continue)

**Constants, initial conditions**	**Value** (min^{-1})	**Ref.**	**Constants, initial conditions**	**Value** (min^{-1})	**Ref.**
$Kcat_{GLUT\ 1}$	69960	15	Kd_{ENO}	0.00048	25
$Kcat_{GLUT\ 2}$	445500	Est	Kd_{PDK}	0.00048	26
$Kcat_{HK}$	12000	5	Kd_{TPI}	0.069	27
$Kcat_{PGI1}$	125400	5	Kd_{LDH}	0.0013	28
$Kcat_{PGI2}$	119586.8	5,22	Kd_{PGCM}	0.00016	29
$Kcat_{PFK\text{-}1}$	61200	5	K_{GLUTh}	0.0001	30
$Kcat_{GAPDH1}$	17400	5	K_{HKh}	0.003	30
$Kcat_{GAPDH2}$	19.81	5,31	K_{PGIh}	0.037	32
$Kcat_{PGK}$	29210	5	$K_{PFK\text{-}1h}$	0.026	32
$Kcat_{PGM1}$	60000	5	K_{PAPDHh}	0.83	33
$Kcat_{PGM2}$	27272.7	5,12	K_{PGKh}	0.3	34
$Kcat_{ENO1}$	11160	5	K_{PGMh}	0.36	35
$Kcat_{ENO2}$	2610.96	5,36	K_{ENOh}	0.23	37
$Kcat_{PDK}$	33	38	K_{PDHh}	0.000017	39
$kcat_{TPI1}$	350000	5	K_{TPIh}	0.3	40
$Kcat_{TPI2}$	7700092.4	5,20	K_{LDHh}	0.003	41
$Kcat_{LDH1}$	122000	19	K_{PGCMh}	0.065	42
$Kcat_{LDH2}$	4900	43	$Kd_{Ac\text{-}CoA}$	10000	Est
$Kcat_{PDH}$	105800	5	Kd_{PDK}	0.00048	26
$Kcat_{PGCM1}$	73700	5	Kd_{TPI}	0.069	27
$Kcat_{PGCM2}$	127220.9	5,22	Kd_{LDH}	0.0013	28
Kd_{GLUT}	0.00016	44	Kd_{PGCM}	0.00016	29
Kd_{HK}	0.002	28	**Constants, initial conditions**	**Value** ($\mu M.min^{-1}$)	**Ref.**
Kd_{PGI}	0.00048	26	$Vm_{GLUT'}$	8000	2
$Kd_{PFK\text{-}1}$	0.00085	26	Vm_{ALDO1}	1028106	5
Kd_{GAPDH}	0.00016	45	Vm_{ALDO2}	832276.3	5,46
Kd_{PGK}	0.0013	28	Vm_{PK}	2819725.8	5
Kd_{PGM}	0.0197	47	Vm_{G6PDH}	120120	5

K_{cat}=catalytic constant, K_m= Michaelis–Menten constant, V_m=maximum velocity, K_d=degradation constant, K_{Eh}=constant of HIF effect on enzyme production, (calculated based on experimental data reported in respective references), 1=in forward direction, 2=in reverse direction. GLUT: glucose transporter, HK: hexokinase, PGI: phosphoglucoisomerase, PFK-1: phosphofructokinase-1, ALDO: aldolase, TPI: triosephosphate isomerase, GAPDH: glyceraldehyde 3-phosphate dehydrogenase, PGK: phosphoglycerate kinase, PGM: phosphoglycerate mutase, ENO: enolase, PK: pyruvate kinase, PDH: pyruvate dehydrogenase, PDK: pyruvate dehydrogenase kinase, PDP: pyruvate dehydrogenase phosphatase, p-PDH: phosphorylated pyruvate dehydrogenase, LDH: lactate dehydrogenase, PGCM: phosphoglucomutase, G6PDH: glucose 6-phosphate dehydrogenase, Eg: extracellular glucose, h: HIF-1, Est: estimated by the proposed model.

References

1 Aiello, D. P., Fu, L., Miseta, A. & Bedwell, D. M. Intracellular glucose 1-phosphate and glucose 6-phosphate levels modulate Ca2+ homeostasis in Saccharomyces cerevisiae. *J. Biol. Chem.* **277**, 45751-45758, doi: 10.1074/jbc.M208748200 (2002).

2 Day, P., Cleal, J., Lofthouse, E., Hanson, M. & Lewis, R. What factors determine placental glucose transfer kinetics? *Placenta* **34**, 953-958, doi:10.1016/j.placenta.2013.07.001 (2013).

3 J John, S. A., Ottolia, M., Weiss, J. N. & Ribalet, B. Dynamic modulation of intracellular glucose imaged in single cells using a FRET-based glucose nanosensor. *Pflugers Archiv.* **456**, 307-322, doi:10.1007/s00424-007-0395-z (2008).

4 Hajjawi, O. S. Glucose transport in human red blood cells. *Am. J. Biomed. Life Sci.* **1**, 44-52, doi:10.11648/j.ajbls.20130103.12 (2013).

5 Albe, K. R., Butler, M. H. & Wright, B. E. Cellular concentrations of enzymes and their substrates. *J. Theor. Biol.* **143**, 163-195, doi:10.1016/S0022 (1990).

6 Doenst, T., Han, Q., Goodwin, G. W., Guthrie, P. H. & Taegtmeyer, H. Insulin does not change the intracellular distribution of hexokinase in rat heart. *Am. J. Physiol. Endocrinol. Metab.* **275**, E558-E567, doi:10.1152/ajpendo.1998.275.4.E558 (1998).

7 Jannaschk, D., Burgos, M., Centerlles, J. J., Ovadi, J. & Cascante, M. Application of metabolic control analysis to the study of toxic effects of copper in muscle glycolysis. *FEBS Lett.* **445**, 144-148, doi:10.1016/s0014-5793(99)00117-9 (1999).

8 Dunaway, G. A., Kasten, T. P., Sebo, T. & Trapp, R. Analysis of the phosphofructokinase subunits and isoenzymes in human tissues. *Biochem. J.* **251**, 677-683, doi:10.1042/bj2510677 (1988).

9 Penhoet, E. E., Kochman, M. & Rutter, W. J. Molecular and catalytic properties of aldolase C. *Biochemistry* **8**, 4396-4402, doi:10.1021/bi00839a026 (1969).

10 Lambeir, A. M. *et al.* The cytosolic and glycosomal glyceraldehyde-3-phosphate dehydrogenase from Trypanosoma brucei: Kinetic properties and comparison with homologous enzymes. *Eur. J. Biochem.* **198**, 429-435, doi:10.1111/j.1432-1033.1991.tb16032.x (1991).

11 Khoroshilova, N. A., Muronetz, V. I. & Nagradova, N. K. Interaction between d-glyceraldehyde-3-phosphate dehydrogenase and 3-phosphoglycerate kinase and its functional consequences. *FEBS Lett.* **297**, 247-249, doi:10.1016/0014-5793(92)80548-U (1992).

12 Fraser, H. I., Kvaratskhelia, M. & White, M. F. The two analogous phosphoglycerate mutases of Escherichia coli. *FEBS Lett.* **455**, 344-348, doi:10.1016/S0014-5793(99)00910-2 (1999).

13 Nakayama, Y., Kinoshita, A. & Tomita, M. Dynamic simulation of red blood cell metabolism and its application to the analysis of a pathological condition. *Theor. Biol. Med. Model.* **2**, 18-28, doi:10.1186/1742-4682-2-18 (2005).

14 Hannaert, V. *et al.* Kinetic characterization, structure modelling studies and crystallization of Trypanosoma brucei enolase. *Eur. J. Biochem.* **270**, 3205-3213, doi:10.1046/j.1432-1033.2003.03692.x (2003).

15 Simpson, I. A., Carruthers, A. & Vannucci, S. J. Supply and demand in cerebral energy metabolism: the role of nutrient transporters. *J. Cereb. Blood Flow Metab.* **27**, 1766-1791, doi:10.1038/sj.jcbfm.9600521 (2007).

16 Boxer, M. B. *et al.* Evaluation of substituted N, N′-diarylsulfonamides as activators of the tumor cell specific M2 isoform of pyruvate kinase. *J. Med. Chem.* **53**, 1048-1055, doi:10.1021/jm901577g (2009).

17 Hucho, F. *et al.* α-Keto acid dehydrogenase complexes: XVII. Kinetic and regulatory properties of pyruvate dehydrogenase kinase and pyruvate dehydrogenase phosphatase from bovine kidney and heart. *Arch. Biochem. Biophys.* **151**, 328-340, doi:10.1016/0003-9861(72)90504-8 (1972).

18 Korotchkina, L. G. & Patel, M. S. Probing the mechanism of inactivation of human pyruvate dehydrogenase by phosphorylation of three sites. *J. Biol. Chem.* **276**, 5731-5738, doi:10.1074/jbc.M007558200 (2001).

19 Pesce, A., Fondy, T. P., Stolzenbach, F., Castillo, F. & Kaplan, N. O. The comparative enzymology of lactic dehydrogenases III. Properties of the H4 and M4 enzymes from a number of vertebrates. *J. Biol. Chem.* **242**, 2151-2167 (1967).

20 Veech, R., Raijman, L., Dalziel, K. & Krebs, H. Disequilibrium in the triose phosphate isomerase system in rat liver. *Biochem. J.* **115**, 837-842 (1969).

21 Grabowska, D. *et al.* A novel mutation in the glucose-6-phosphate dehydrogenase gene in a subject with chronic nonspherocytic hemolytic anemia—characterization of enzyme using yeast expression system and molecular modeling. *Blood Cells. Mol. Dis.* **32**, 124-130, doi:10.1016/j.bcmd.2003.11.001 (2004).

22 Kashiwaya, Y. *et al.* Control of glucose utilization in working perfused rat heart. *J. Biol. Chem.* **269**, 25502-25514 (1994).

23 Ramström, H. *et al.* Properties and regulation of the bifunctional enzyme HPr kinase/phosphatase in Bacillus subtilis. *Journal of Biological Chemistry* **278**, 1174-1185, doi:10.1074/jbc.M209052200 (2003).

24 Qutub, A. A. & Popel, A. S. A computational model of intracellular oxygen sensing by hypoxia-inducible factor HIF1α. *Journal of cell science* **119**, 3467-3480, doi:10.1242/jcs.03087 (2006).

25 Tiainen, M., Roine, R. O., Pettilä, V. & Takkunen, O. Serum neuron-specific enolase and S-100B protein in cardiac arrest patients treated with hypothermia. *Stroke* **34**, 2881-2886, doi:10.1161/01.STR.0000103320.90706.35 (2003).

26 Illg, D. & Pette, D. Turnover Rates of Hexokinase I, Phosphofructokinase, Pyruvate Kinase and Creatine Kinase in Slow-Twitch Soleus Muscle and Heart of the Rabbit. *Eur. J. Biochem.* **97**, 267-273, doi:10.1111/j.1432-1033.1979.tb13111.x (1979).

27 Alvarez, M. *et al.* Triose-phosphate Isomerase (TIM) of the Psychrophilic BacteriumVibrio marinus KINETIC AND STRUCTURAL PROPERTIES. *Journal of Biological Chemistry* **273**, 2199-2206, doi:10.1074/jbc.273.4.2199 (1998).

28 J Jones, G. M. & Mayer, R. Degradation of glucose-metabolizing enzymes in the rat small intestine during starvation. *Biochem. J.* **132**, 657-661, doi:10.1042/bj1320657a (1973).

29 Wang, Y. & Zhang, Y. H. A highly active phosphoglucomutase from Clostridium thermocellum: cloning, purification, characterization and enhanced thermostability. *Journal of applied microbiology* **108**, 39-46, doi:10.1111/j.1365-2672.2009.04396.x (2010).

30 Shinohara, Y., Hino, M., Ishida, T., Yamanaka, Y. & Terada, H. Growth condition-dependent synchronized changes in transcript levels of type II hexokinase and type 1 glucose transporter in tumor cells. *Biochimica et Biophysica Acta (BBA)-Molecular Cell Research* **1499**, 242-248, doi:10.1016/S0167-4889(00)00125-7 (2001).

31 Boschi-Muller, S., Azza, S., Pollastro, D., Corbier, C. & Branlant, G. Comparative Enzymatic Properties of GapB-encoded Erythrose-4-Phosphate Dehydrogenase of Escherichia coliand Phosphorylating Glyceraldehyde-3-phosphate Dehydrogenase. *J. Biol. Chem.* **272**, 15106-15112, doi:10.1074/jbc.272.24.15106 (1997).

32 Bousquet, P. A. *et al.* Hypoxia strongly affects mitochondrial ribosomal proteins and translocases, as shown by quantitative proteomics of HeLa cells. *International journal of proteomics* **2015**, 678527-678536, doi:org/10.1155/2015/678527 (2015).

33 Escoubet, B., Planès, C. & Clerici, C. Hypoxia increases glyceraldehyde-3-phosphate dehydrogenase transcription in rat alveolar epithelial cells. *Biochem. Biophys. Res. Commun.* **266**, 156-161, doi:10.1006/bbrc.1999.1798 (1999).

34 Lam, W., Bussom, S. & Cheng, Y.-C. Effect of hypoxia on the expression of phosphoglycerate kinase and antitumor activity of troxacitabine and gemcitabine in non-small cell lung carcinoma. *Molecular cancer therapeutics* **8**, 415-423, doi: 10.1158/1535-7163.MCT-08-0692 (2009).

35 Takahashi, Y., Takahashi, S., Yoshimi, T. & Miura, T. Hypoxia-induced expression of phosphoglycerate mutase B in fibroblasts. *European journal of biochemistry* **254**, 497-504, doi:10.1046/j.1432-1327.1998.2540497.x (1998).

36 Pietkiewicz, J., Bednarz-Misa, I., Jermakow, K. & Gamian, A. Enolase from Klebsiella pneumoniae and human muscle cells ii. kinetic parameters and sensitivity to fluoride and phosphate inhibitors. *Advances in Clinical and Experimental Medicine* **18**, 221-233 (2009).

37 Sedoris, K. C., Thomas, S. D. & Miller, D. M. Hypoxia induces differential translation of enolase/MBP-1. *BMC Cancer* **10**, 157-170, doi:10.1186/1471-2407-10-157 (2010).

38 Tovar-Méndez, A., Hirani, T. A., Miernyk, J. A. & Randall, D. D. Analysis of the catalytic mechanism of pyruvate dehydrogenase kinase. *Archives of biochemistry and biophysics* **434**, 159-168, doi:10.1016/j.abb.2004.10.017 (2005).

39 Kim, J.-w., Tchernyshyov, I., Semenza, G. L. & Dang, C. V. HIF-1-mediated expression of pyruvate dehydrogenase kinase: a metabolic switch required for cellular adaptation to hypoxia. *Cell metabolism* **3**, 177-185, doi:10.1016/j.cmet.2006.02.002 (2006).

40 Gess, B., Hofbauer, K.-H., Deutzmann, R. & Kurtz, A. Hypoxia up-regulates triosephosphate isomerase expression via a HIF-dependent pathway. *Pflügers Archiv* **448**, 175-180, doi:10.1007/s00424-004-1241-1 (2004).

41 Firth, J. D., Ebert, B. L. & Ratcliffe, P. J. Hypoxic regulation of lactate dehydrogenase A Interaction between hypoxia-inducible factor 1 and cAMP response elements. *Journal of Biological Chemistry* **270**, 21021-21027, doi:10.1074/jbc.270.36.21021 (1995).

42 Pelletier, J. *et al.* Glycogen synthesis is induced in hypoxia by the hypoxia-inducible factor and promotes cancer cell survival. *Frontiers in oncology* **2**, 18-27, doi:10.3389/fonc.2012.00018 (2012).

43 Gomez, M. S. *et al.* Substrate and cofactor specificity and selective inhibition of lactate dehydrogenase from the malarial parasite P. falciparum. *Mol. Biochem. Parasitol.* **90**, 235-246, doi:10.1016/s0166-6851(97)00140-0 (1997).

44 Heilig, C. *et al.* Implications of glucose transporter protein type 1 (GLUT1)-haplodeficiency in embryonic stem cells for their survival in response to hypoxic stress. *The American journal of pathology* **163**, 1873-1885, doi:10.1016/S0002-9440(10)63546-8 (2003).

45 Kuehl, L. & Sumsion, E. N. Turnover of several glycolytic enzymes in rat liver. *Journal of Biological Chemistry* **245**, 6616-6623 (1970).

46 Malay, A. D., Procious, S. L. & Tolan, D. R. The temperature dependence of activity and structure for the most prevalent mutant aldolase B associated with hereditary fructose intolerance. *Arch. Biochem. Biophys.* **408**, 295-304, doi:10.1016/s0003-9861(02)00546-5 (2002).

47 Nairn, J. *et al.* The use of mass spectrometry to examine the formation and hydrolysis of the phosphorylated form of phosphoglycerate mutase. *FEBS Lett.* **359**, 192-194, doi:0.1016/0014-5793(95)00044-a (1995)
